# The use of technology in cancer prehabilitation: a systematic review

**DOI:** 10.3389/fonc.2024.1321493

**Published:** 2024-04-19

**Authors:** San San Tay, Fuquan Zhang, Edmund Jin Rui Neo

**Affiliations:** Department of Rehabilitation Medicine, Changi General Hospital, Singapore, Singapore

**Keywords:** cancer prehabilitation, technology, feasibility, effectiveness, telemedicine, wearables

## Abstract

**Aim:**

This review aimed to evaluate the effectiveness and feasibility of cancer prehabilitation programs delivered through technological enablers compared to conventional face-to-face interventions.

**Methods:**

A systematic review was conducted, searching PubMed, Embase, and CINAHL for studies published from inception to February 6, 2024. Studies were included if they involved adult cancer patients in primary research, utilized technology for prehabilitation, and assessed functional, psychological, and quality of life outcomes.

**Results:**

Sixteen studies were included, encompassing wearables, apps, teleprehabilitation, and virtual reality. All studies reported feasibility, but challenges included technical issues, lack of supervision, and non-compliance. Effectiveness depended on intervention rigor and technology type. Wearables offered objective monitoring but faced compliance issues. Videoconferencing provided supervision and could mitigate compliance concerns. Multimodal programs and intervention-specific outcome measures were recommended.

**Conclusion:**

Technology-based prehabilitation programs seem feasible, but effectiveness depends on intervention design and technology employed. Future research should focus on developing robust evidence to guide clinical practice and explore the potential of integrated technological solutions.

**Systematic review registration:**

PROSPERO, identifier CRD42022376028.

## Introduction

Cancer prehabilitation, defined as interventions delivered prior to the commencement of acute cancer treatments (surgery, chemotherapy, radiation therapy, or immunotherapy) (1), has seen an increase in the use of technology during the COVID-19 pandemic. This aligns with broader healthcare trends towards telemedicine and telehealth applications (2, 3). While some countries had established telehealth services pre-pandemic, facilitating easier scaling-up during the crisis (3), cancer prehabilitation also underwent adaptations (4, 5). This increased utilization of technology warrants a review of the different modes employed, their effectiveness, and feasibility.

Cancer prehabilitation programs can encompass single or multimodal interventions including exercise, nutritional guidance, and psychological support (6, 7). Specific diagnostic groups may benefit from additional targeted interventions such as respiratory muscle training and breathing exercises for pre-thoracic surgery or pelvic floor exercises and sexual well-being support in patients with prostate cancer (8, 9). Research also includes studies focusing solely on exercise-based (10) or nutritional interventions (11).

The pandemic necessitated adaptations to traditional in-person delivery models, shifting interactions to phone calls or web conferencing (12–14). Other approaches have implemented adaptive case management platforms integrated with electronic health records (15). Notably, a telehealth-delivered home-based prehabilitation program adapted from a face-to-face model has demonstrated feasibility and effectiveness (16). This raises questions about exploring other existing technologies, such as wearables and robotics, in cancer prehabilitation research.

Despite the increased use of technology in cancer prehabilitation, information on its efficacy, acceptability, and feasibility compared with conventional face-to-face interventions remains limited. While knowledge on telerehabilitation has existed for two decades (17), teleprehabilitation and other technology-enabled applications for remote prehabilitation are more recent developments (12). A systematic review of the cost-utility and cost-effectiveness of telemedicine, electronic, and mobile health systems yielded mixed results (18). Further understanding of diverse technology deployments and their impact on efficacy, acceptability, and feasibility is crucial before their widespread adoption.

Accessibility is another key consideration, particularly in areas with limited healthcare access due to geographical barriers or resource constraints. For patients already owning smartphones, technology-based interventions offer the potential to improve healthcare accessibility and reduce travel time, costs, and work disruptions (19, 20).

To date, no systematic review has comprehensively assessed the effectiveness and feasibility of cancer prehabilitation programs leveraging technological enablers. This review aims to address this gap.

The primary objective was to evaluate the effectiveness of cancer prehabilitation delivered through technological enablers compared with conventional face-to-face interventions or standard care. The secondary objective was to evaluate the feasibility of these technology-based programs.

## Methods

### Inclusion criteria

This systematic review included studies that met the following criteria.

1. Primary research (randomized and non-randomized experimental trials, cohort, or case-control studies).
2. Studied cancer prehabilitation as an intervention.
3. Applied cancer prehabilitation prior to surgical as well as non-surgical intervention (for example chemotherapy, radiation therapy, immunotherapy, hormonal therapy).
4. Utilised technology such as trackers, apps, telehealth, virtual or online platforms and robotic devices for cancer prehabilitation.
5. The participants were aged 18 years or older.

Both single and multimodal prehabilitation models were included due to the focus on technology use.

### Exclusion criteria:

Studies were excluded if they were:

1. Presented as conference proceedings, poster abstracts, case reports or study protocols.
2. Animal studies.
3. Rehabilitation instead of prehabilitation studies.

### Study registration

This protocol was developed according to the Preferred Reporting Items for Systematic Reviews and Meta-Analyses (PRISMA) Protocols. This study was registered with the PROSPERO database, protocol number CRD42022376028.

### Search methods

#### Population

Adult patients aged 18 years or older with cancer.

#### Interventions

The use of telehealth, wearables, smart phone applications, virtual or online platforms, robotics and virtual reality in the application of cancer prehabilitation, prior to any cancer surgery, chemotherapy, radiation therapy, immunotherapy, or hormonal therapy.

#### Comparison

Patients who received conventional face-to-face prehabilitation or standard care.

#### Outcomes

Functional, psychological, and quality of life domains.

Search Terms:

Searches were performed on PubMed, Embase, and the Cumulative Index to Nursing and Allied Health Literature (CINAHL) from inception until 6 Feb 2024.

The search strategy included “cancer” AND “prehabilitation” AND “technology”. We combined synonyms and MeSH terms with the “OR” operator (**Table 1**). This strategy was employed for PubMed and was adapted for use with other databases. In addition, we checked the reference lists of all the included trials and relevant systematic reviews to identify potentially eligible studies.

**Table 1:** Search strategy by database and type of search.

Database	Search Terms	Hits
Embase (Keyword)	(cancer OR malignancy OR tumour OR neoplasm OR tumor OR carcinoma) AND ((Preoperative AND rehabilitation) OR prehab OR (preoperative AND exercise)) AND (technology OR digital OR virtual OR robotics OR tele*)	**525**
PubMed (Keyword)	(cancer OR malignancy OR tumour OR neoplasm OR tumor OR carcinoma) AND ((Preoperative AND rehabilitation) OR prehab OR (preoperative AND exercise)) AND (technology OR digital OR virtual OR robotics OR tele*)	**379**
CINAHL (Keyword)	(cancer OR malignancy OR tumour OR neoplasm OR tumor OR carcinoma) AND ((Preoperative AND rehabilitation) OR prehab OR (preoperative AND exercise)) AND (technology OR digital OR virtual OR robotics OR tele*)	**21**

All titles and abstracts were reviewed for relevance. Full-text articles were read when they were eligible or when eligibility was unclear. Two independent reviewers (SST and FQZ) screened all articles to assess their eligibility. A third reviewer (EN) was available for discussion in case of disagreement.

### Types of outcome measures

#### Primary outcomes

Physical function including the 6 min walk test (6MWT), time up and go test (TUG), and the 30 seconds sit-to-stand test (30 sec STS).

Psychological and quality of life outcomes were assessed using measures such as the Hospital Anxiety and Depression Scale (HADS) (21), EuroQol-5D (EQ5D) (22), and European Organization for Research and Treatment of Cancer Quality of Life Questionnaire C-30 (EORTC QLC-C30) (23).

#### Secondary outcomes

Outcome indicators such as length of stay, postsurgical complications, other patient-reported outcomes (PROM), readmission rates, safety, and feasibility will be reported if available.

### Data extraction and outcomes

Data extraction of the included articles was conducted by two reviewers: SST and FQZ. FQZ collated the relevant features of each study using data extraction sheets and compiled them in a computerised database, which was counterchecked by SST for accuracy.

National Institutes of Health (NIH) quality assessment tools were deployed, and tailored quality assessment was applied separately for controlled interventions and for before-after (pre-post) studies with no control group (24). NIH tools facilitate a comprehensive assessment of a range of aspects of research quality, such as randomization, allocation, blinding, inter-group demographics, drop-outs, intervention adherence, missing data, and data analysis.

Each reviewer rated the studies as good, fair, or poor. No official published system is available for denoting the overall quality of NIH tools. As such, we deemed the individual quality indicators to have similar weights for internal validity and ascribed overall quality through cumulative scores. We followed this application in another review project (25). A cumulative score of <50% quality indicators present denoted a poor-quality study (significant risk of bias), while >80% present denoted a good-quality study (least risk of bias). A “fair” study (50-80% quality indicators present) would be susceptible to some bias deemed insufficient to invalidate its results. The range can be broad and each study would have its strength and weaknesses.

Two reviewers (SST and FQZ) were involved in the quality assessment and disagreements were escalated to a third reviewer (EN).

### Statistical analysis

Data were qualitatively synthesized to evaluate changes in the three primary outcome domains (physical, psychological, and quality of life) between the pre- and post-intervention phases. Feasibility and safety of the interventions were also synthesized and reported. Attempts were made to contact the study authors if there were any cases of missing data. This study conformed to all Preferred Reporting Items for Systematic Reviews and Meta-Analyses (PRISMA) guidelines and reported the required information accordingly.

## Results

**Figure 1:**
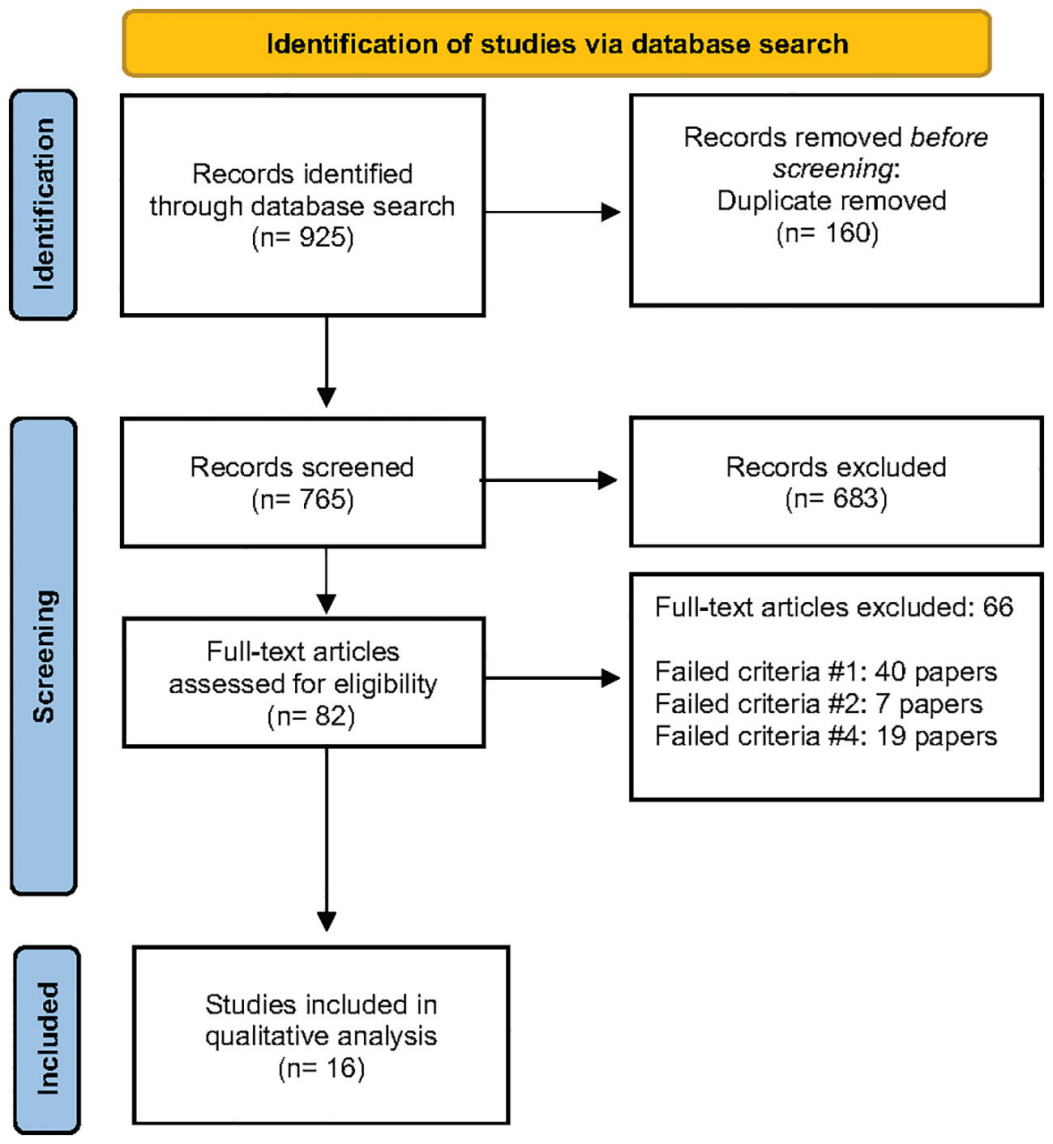
PRISMA flow diagram.

A total of 765 records were screened after the computer-assisted removal of duplicates. After screening the titles, 115 were chosen to be screened for abstracts, of which 82 were chosen to have the full-text articles assessed for eligibility. After a full-text review, 66 studies were excluded, and 16 were included in the qualitative synthesis. Forty papers failed criteria #1, (14, 19, 26–63), seven papers failed criteria #2, (64–70) and 19 papers failed criteria #4, (12, 17, 71–87) and all were excluded from the systematic review (**Supplementary Table 1**). The PRISMA flow diagram is found in **Figure 1**.

### Methodological quality

Two studies initially categorized as cohort studies were reclassified as pre-post due to clear interventions with pre- and post-intervention data (88, 89). Most studies had small participant numbers (< 50), except for one (16) with 100 participants.

Among the 16 included studies, four were controlled intervention studies [one rated good (90), two fair (91, 92), and one poor (93)]. Twelve were pre-post studies [ten were fair with some bias risk (16, 88, 94–101) and two with significant risk (89, 102)]. Due to their heterogeneity and limited large-scale studies, three poor-quality studies were included in the qualitative analysis (**Table 2**).

**Table 2:** Study quality assessed by quality assessment tools for controlled intervention and pre-post studies.

Authors	Items of Quality Assessment Tool for Controlled Intervention Studies																	
	1	2	3	4	5	6	7	8	9	10	11	12	13	14	Total score	Poor <8	Fair 8-11	Good 12-14
Czech et al., 2023 (92)	Y	Y	Y	N	NR	Y	Y	Y	Y	NR	Y	N	Y	Y	10/14		✔	
Patel et al., 2023 (90)	Y	Y	Y	Y	Y	Y	Y	Y	Y	Y	Y	N	Y	Y	13/14			✔
Rodriguez et al., 2023 (93)	Y	NR	N	N	NR	Y	N	N	N	CD	Y	N	Y	Y	5/14	✔		
Waller et al., 2022 (91)	Y	Y	Y	Y	NR	Y	Y	Y	Y	Y	Y	N	Y	Y	12/14		✔	
Authors	Items of Quality Assessment Tool for Pre-post Studies																	
	1	2	3	4	5	6	7	8	9	10	11	12			Total score	Poor <7	Fair 7-9	Good 10-12
Bruns et al., 2019 (96)	Y	Y	Y	Y	N	Y	Y	N	Y	N	N	Y			8/12		✔	
Chmelo et al., 2022 (88)	Y	Y	Y	N	Y	Y	Y	N	NR	N	Y	NA			7/12		✔	
Drummond et al., 2022 (98)	Y	Y	Y	Y	N	Y	Y	NR	Y	Y	N	NA			8/12		✔	
Finley et al., 2020 (102)	Y	Y	Y	Y	N	Y	Y	NA	N	N	N	NA			6/12	✔		
Finley et al., 2021 (94)	Y	Y	Y	Y	N	Y	Y	N	Y	Y	Y	NA			9/12		✔	
Franssen et al., 2022 (97)	Y	Y	Y	Y	N	Y	Y	N	Y	Y	N	Y			9/12		✔	
Kadiri et al., 2019 (89)	Y	Y	Y	NR	N	Y	Y	Y	N	N	N	NA			6/12	✔		
Lorca et al., 2023 (100)	Y	Y	Y	N	N	Y	Y	N	N	Y	Y	NA			7/12		✔	
Moorthy et al., 2023 (99)	Y	Y	Y	Y	N	Y	Y	NR	Y	Y	Y	NA			9/12		✔	
Piche et al., 2023 (101)	Y	Y	N	Y	N	Y	Y	N	Y	Y	NR	NA			7/12		✔	
Piraux et al., 2020 (95)	Y	Y	Y	Y	N	Y	Y	N	N	Y	Y	Y			9/12		✔	
Wu et al., 2021 (16)	Y	Y	Y	Y	N	Y	Y	N	N	Y	N	NA			7/12		✔	

Total score, number of Yes; NA, not applicable; NR, not reported; N, not present; Y, present.

### Included studies

Of the 16 included studies, they were divided into 4 groups of technological enablers, namely wearables, apps, teleprehabilitation and virtual reality (**Table 3**).

**Table 3:** Outcome measures and results.

Authors	No. of Patients; Gender Age, mean (years) BMI, mean (kg.m-2) Type of cancers	Type of Study Interventions Used Technological Enabler Used	Feasibility/Outcome Measures	Results
Wearables				
Chmelo (2022) UK (88)	39; Male 33 Female 6 68 27.3 Locally advanced oesophageal and gastric Ca	• Prospective, single-center feasibility study, single arm • Interventions: aerobic training, strength training • Technology: Pedometer	• Demonstrated feasibility & acceptability. 72.4% recruitment. • Outcome measures: Recruitment rate, completion rate, engagement with program, exercise compliance	• Almost everyone (98.7%) wore their pedometer and recorded data. Everyone (100.0%) participated in the weekly telephone consultations. 70.2% completed exercise sessions, & 69.4% completed strengthening exercises. • Improvement in physical function, decreased fatigue, reduced nausea, & improved appetite reported. Mean score of global health status improved. No adverse events reported. • Financial support: The Jon Moulton Charitable Foundation, UK; The Sir Bobby Robson Foundation, Newcastle-upon-Tyne, UK.
Finley (2020) UK (102)	28; Male 12 Female 16 67.3 BMI not provided Lung cancer	• Single arm, pre-post feasibility study (pilot) • Interventions: aerobic training • Technology: Wrist-worn smartwatch (Garmin Vivoactive HR) connected to smartphone	• Demonstrated feasibility. 79% participants completed pre-op study. 71% enrolled participants successfully synchronized device. • Outcome measures: Feasibility, acceptability, and perceived utility of wearable fitness device with exercise prescription.	• Highest retention in the pre-operative period, with a decline in participation after surgery. • Patient’s satisfaction: 36% liked the device. 79% of participants disliked some aspect of the fitness device, and 29% didn’t understand how to use it properly. • Financial support: Population Sciences Developmental Pilot Fund award from the Norris Cotton Cancer Center, Lebanon, NH; National Institute On Aging (NIA); National Center for Advancing Translational Sciences (NCATS); Dartmouth Health Promotion and Disease Prevention Research Center; National Institute of Mental Health
Finley (2021) UK (94)	18; Male 8 Female 10 68.2 BMI not provided Stage 1-3 Lung cancer	• Single arm pre-post proof-of- concept study • Interventions: aerobic training • Technology: Wrist-worn smartwatch Garmin fitness device	• 17 out of 18 participants completed 6MWT at baseline & day of surgery. • Outcome measures: Level of MVPA and change in aerobic capacity.	• Nil significant 6MWT increase. 47% experienced MCID of 14m or more. • Patient’s satisfaction not reported. • Financial support: Population Sciences Developmental Pilot Fund award from the Norris Cotton Cancer Center, Lebanon, NH; National Institute On Aging (NIA); National Center for Advancing Translational Sciences (NCATS); Dartmouth Health Promotion and Disease Prevention Research Center; National Institute of Mental Health
Patel (2023) Canada (90)	95; Male 40 Female 55 67.24 28.12 Early-stage non-small cell lung cancer	• Single-site, blinded, two-arm, parallel- group RCT • Interventions: Breathing exercises, aerobic exercises, nutrition, education • Technology: Fitbit activity tracker	• 93.1% enrolled patients completed trial. High eligibility & completion rate. • Outcome measures: EQ5D5L, Length of stay, complication rates, QoL.	• Improvement in EQ-5D-5L relating mobility (p=0.008) & pain/discomfort (p<0.001). Length of stay decreased. Nil statistical differences in intraop complications incidence. Nil adverse events reported. • Patient’s satisfaction: 96% patient would continue using activity tracker. • Financial support: Hamilton Academic Health Sciences Organization AFP Innovation Grant
Rodriguez (2023) USA (93)	83; Male 31 Female 52 62.0 (control), 62.9 (intervention) BMI not provided Pancreatic cancer	• Prospective, randomized single-center trial • Interventions: daily step counts • Technology: Fitbit devices	• 55% patient completed study. • Outcome measures: Post-op complications	• Nil statistical difference in rate of post-op complications. • Patient’s satisfaction not reported. • Financial support: The Foundation for Barnes Jewish Hospital & BJC Health Systems Innovation Lab
Waller (2022) UK (91)	22; Male 11 Female 11 61 (control), 55.5 (intervention) 30 (control), 27.8 (intervention) Major abdominal cancer surgery	• Single-blind pilot study RCT • Interventions: aerobic training, resistance exercises, nutrition, education, mental health support • Technology: Wrist-worn smartwatch connected to smartphone application	• 67% recruitment. High compliance with wearing the device and participating in the exercises. • Outcome measures: Levels of physical activity, functional walking capacity (6MWT), body weight changes, psychological well-being (HADS).	• Intervention group engaged in more daily moderate intensity exercise (p=0.063) & vigorous physical activity (p=0.022). Greater improvements in 6MWT distance (p=0.014). HADS scores remained unchanged. • Patient’s satisfaction: 100% satisfaction with prehab program & exercises. 90% agreement that the smart device motivated physical activity. • Financial support: Charity Pseudomyxoma Survivor
Apps				
Bruns (2019) Netherlands (96)	14; Male 5 Female 9 79 25 Colorectal cancer	• Feasibility study • Interventions: aerobic training, strength training, nutrition, education • Technology: Fit 4 Surgery TV - Device prototype developed solely for the study. Digital activating companion, voice indicates activity- breakfast, snack, or exercise	• Demonstrated feasibility & acceptability. 100% Participation rate for recipe prep, 93% of exercises days were engaged. 86% clear user interface • Outcome measures: Feasibility.	• Improvements in functional performances at baseline and post intervention (Fried score and SPPB) • Patient’s satisfaction: Overall increase in quality of life. Many patients would like to keep the device and use them after discharge from hospital. • Financial support: Nil
Kadiri (2019) UK (89)	31; gender not provided 64 25.7 Lung cancer	• Cohort study/Feasibility study • Interventions: aerobic training & strengthening based on ‘Rehabilitation for Operated lung Cancer (ROC)’ Programme, education • Technology: Fit 4 Surgery app - Developed for Ipad mini 2 cellular, with blue tooth enabled pulse oximeter & wireless feedback to researchers. SpO2 and HR monitor	• Demonstrated feasibility. 32% of patients did not use the app post- surgery. For patients who had attended classes pre-op, 79% did not attend classes post-op. Dropout rate of app users were lower than those attending physical classes. • Outcome measures: QoL, Inpatient length of stay, rate of postoperative pulmonary complication (PPC), ITU admissions, hospital length of stay & 30-readmission.	• App patients had shorter wait time to surgery (24 vs 45 days) but completed more exercise sessions than class patients (2 vs 9 sessions). They completed more sessions post-surgery. App group improved incremental shuttle walk test distance (p < 0.05). No comparison between 2 groups for LOS, PPC, ITU admissions, 30-day readmissions. • Patient’s satisfaction: EORTC QLQ-Global Health score at 5 months for the app group improved significantly and returned to baseline. • Financial support: Health Foundation Grant
Piraux E (2020) Belgium (95)	23; Male 16 Female 7 61.7 25.6 Esophagogastric cancer	• Feasibility study • Interventions: aerobic & resistance training, inspiratory muscle training • Technology: Virtuagym fitness app - Personalized account consists of exercise schedule, description of exercises, email tab. Website- set of exercise videos	• Demonstrated feasibility & acceptability. 96% Recruitment & retention rates. 77% & 68% aerobic/resistance training rates & respiratory muscle training attendance rates respectively. • Outcome measures: Primary: recruitment rate, retention rate, attendance, exercise-related adverse events, patient satisfaction. Secondary: Functional exercise capacity, CRF, QoL, anxiety & depression.	• Significant improvement in fatigue, QOL, physical well- being, emotional well-being & anxiety. No adverse events reported. • Patient’s satisfaction: Median satisfaction score of 9.0 (8.0 to 9.9) out of 10-point scale. • Financial support: Fonds National de la Recherche Scientifique (FRIA-FNRS); The Institut de Recherche Expérimentale et Clinique (Université catholique de Louvain, Brussels, Belgium).
Telemedicine				
Drummond (2022) Canada (98)	10; Male 8 Female 2 68 25.5 Elective thoracic and abdominal cancers	• Retrospective pilot cohort study • Interventions: aerobic training, strength training, nutrition, education • Technology: Tablet & wearable device to facilitate communication & data collection. Recorded videos on tablet, videos on nutrition optimization, relaxation exercises. Exercise monitored via tele-conferencing platform. Dietician & psychologist not on tech platform	• NA. • Outcome measures: Completion rate, adverse events, drop-outs and exercise metrics, patients’ experience.	• Increased daily step count (p<0.001), increased duration of exercise per week (p=0.093). Median duration of participation 9.5 weeks. • Patient’s satisfaction: 96% satisfied with program, average perceived usefulness score of 88%. • Financial support: Nil
Franssen (2022) Netherlands (97)	11; Male 6 Female 5 74 29.1 Colorectal cancer	• One-arm pilot Feasibility study • Interventions: aerobic training, nutrition, education • Technology: mobile phone application	• Demonstrated feasibility. 81% participation rate • Outcome measures: Feasibility (determined by participation rate, adverse events, adherence, drop- out rates, retention rates), Patients’ experience.	• Adherence to exercise program’s frequency, intensity and time was 91%, 84%, & 100% respectively. Time to exhaustion improved from median score of 317 seconds to 412 seconds (p = 0.24). Median number of repetitions on the 30-s chair-stand test improved (p = 0.01). No adverse events reported. • Patient’s satisfaction: Most participants felt the tele- prehab program prepared them well for the surgery. Reported usefulness of the smart phone application and knew independent app use after short introduction. • Financial support: Research & Innovation fund VieCuri Medical Center and the National Fund against Cancer (Nationaal Fonds tegen Kanker).
Lorca (2023) Chile (100)	57; Male 29 Female 28 68.8 26.13 Colorectal cancer	• Descriptive, longitudinal retrospective study • Interventions: aerobic training, resistance exercises, balance & proprioception training, education • Technology: videoconferencing through Zoom, smartphone video call	• 53% retention rate. • Outcome measures: Barthel index score, BFI, FSTST, STST 1 min	• Improvements in BFI, FSTST & STS 1-min scores after intervention (p<0.01). Nil differences in Barthel index score. No adverse events recorded. • Patient’s satisfaction: Overall satisfied in terms of usefulness, duration of sessions, content, clarity, comfort, support & safety. • Financial support: Nil
Moorthy (2023) UK (99)	57; Male 43 Female 14 65 (in-person), 67.4 (digital) BMI not provided Oesophageal cancer	• Feasibility study • Interventions: aerobic training, strength training, nutrition, education, mental health support • Technology: Teleconferencing via Digital Prehabilitation Service (DPS) health app	• Demonstrated feasibility. 75% recruitment rate, 84% completion & 86% compliance. • Outcome measures: feasibility & compliance, STS, IPAQ, Emotional Distress Scale, post- operative complication rate, length of hospital stay	• STS improved (p=0.02). No changes in mean self- reported physical activity (p=0.64) or average step count per day (p=0.55). Significant drop in distress (p=0.04). No significant changes in anxiety (p=0.22) & depression (p=0.41). No difference in post-op complication rate and length of hospital stay. • Patient’s satisfaction not reported. • Financial support: Innovate UK, NIHR Imperial Biomedical Research Centre
Piche (2023) Canada (101)	25; Male 5 Female 20 60.2 27.9 Breast cancer, prostate cancer	• Prospective, single-group, pragmatic feasibility study • Interventions: aerobic training, strength training, balance training, education, nutrition, mental health support • Technology: videoconferencing technology with Zoom software.	• Demonstrated feasibility. 92.6% enrollment with 96% retention rate. Most participants completed online questionnaires & telehealth fitness assessment. • Outcome measures: Feasibility & acceptability, 2-min step test, 30s STS, HADS, EORTC-QLQ-C30, MOS-SSS	• Improved physical functional capacity based on 2-min step test (p=0.005), 30s STS (p=0.011), & volume of moderate intensity physical activity (p<0.001). Nil significant changes in HADS, EORTC-QLQ-C30 & MOS-SSS. • Patient’s satisfaction: All participants were satisfied. • Financial support: Universite de Montreal, Programme de soutien aux projets technosociaux innovants
Wu (2021) UK (16)	100 participants, 66 completed questionaire; Male 34 Female 32 67 27.9 Colorectal, urology, breast, cardiothoracic cancers, for surgery & non-surgical treatment	• Prospective, observation study • Interventions: aerobic exercises, resistance exercises, inspiratory muscle training (lung & breast cancer patients), nutrition, education, mental health support • Technology: Teleconferencing	• Demonstrated feasibility & acceptability. 72.4% recruitment. • Outcome measures: Feasibility.	• Improved self-perceived health (p = 0.001) and fatigue (p = 0.000) via EQ5D3L and FACIT-F scale respectively. • Patient’s satisfaction: Positive patient experience overall. • Financial support: Kent and Medway Cancer Alliance
Virtual reality				
Czech (2023) Poland (92)	16; Male 0 Female 16 59.55 (control), 50.59 (intervention) 23.83 (control), 26.61 (intervention) Malignant breast cancer	• Pragmatic pilot study • Interventions: Mental health support. Exercises not described. • Technology: Virtual Therapeutic Gargen (VRTierOne) virtual reality (VR)-based therapy	• NA. • Outcome measures: IPAQ, BDI, Mini-MAC, Pittsburgh Sleep Quality Index	• Reduction in destructive style of coping (p=0.003) & increase in coping (p=0.044) with disease after therapy. Reduction in distress and anxiety (p=0.02). • Patient’s satisfaction: Positive patient experience overall. • Financial support: Nil

Six studies involved wearables such as a wrist-worn smartwatch (90, 91, 93, 94, 102) and pedometers (88). Three studies involved smart applications, such as Fit 4 Surgery TV (96), which was a prototype designed solely for the study; an exercise app (89); or a virtual gym app (95) that consisted of an exercise schedule, description of exercises, and an email tab. The exercise videos were hosted on a website.

Teleprehabilitation was conducted in 7 studies. One involved a tablet (98) and wearable device to facilitate communication and data collection. Recorded videos of nutrition and relaxation techniques were available on the tablet, whereas exercise was monitored on a teleconferencing platform. Another study conducted home physical exercise training using a mobile phone application with a heart rate monitor (97). The third study involved telehealth that delivered a multimodal home-based prehabilitation program involving exercise, nutrition, medical optimization and psychological support (16). Another study utilized video-conferencing for teleprehabilitation via the zoom platform or a videocall on the smart phone (100). The fifth involved tele-conferencing either through an app or on the web coupled with a wearable tracker for remote monitoring (99). Finally, group exercises and educational programs were carried out via videoconferencing (101).

Teleprehabilitation is broadly encompassing. In most of these studies, it involves the provision of digital information or material, and there is a feedback loop, be it through emails or phone calls. In most cases, there may be more than 1 type of technology provided, eg app and wearable (98). It is noteworthy that teleprehabilitation that provided supervision through videoconferencing trended towards improvements in physical outcome measures (99–101) (**Table 4**).

**Table 4:** Technology and its effectiveness. .

Authors	Technological Enabler Used					Interventions							
	Wearables	Apps	Telehealth		VR	Exercise	Improvements	Nutrition	Improvements	Mental care	Improvements	Education	Improvements
			Video conferencing	Supervised exercises									
Chmelo (2022) UK [88]	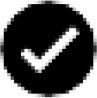					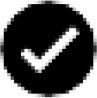							
Finley (2020) UK [102]	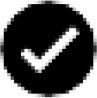					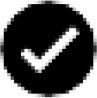							
Finley (2021) UK [94]	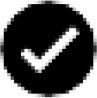					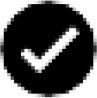							
Patel (2023) Canada [90]	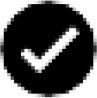					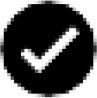	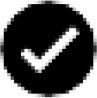 **a**	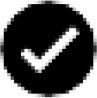				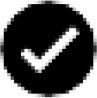	
Rodriguez (2023) USA [93]	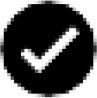					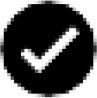		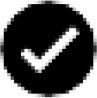					
Waller (2022) UK [91]	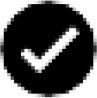					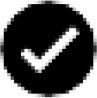	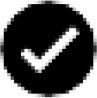 **b**	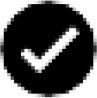		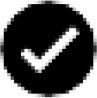		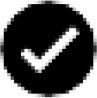	
Bruns (2019) Netherlands [96]		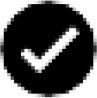				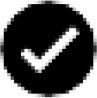		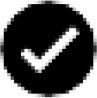				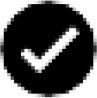	
Kadiri (2019) UK [89]		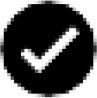				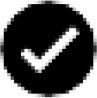	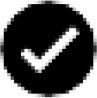 **c**					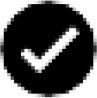	
Piraux E (2020) Belgium [95]		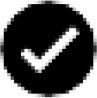	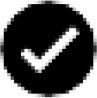			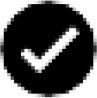	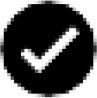 **d**						
			N	N									
Drummond (2022) Canada [98]	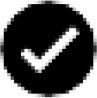		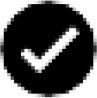			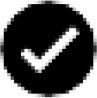	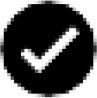 **e**	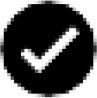				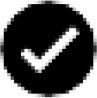	
			N	N									
Franssen (2022) Netherlands [97]	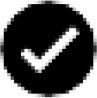		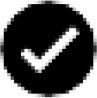			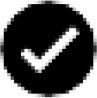	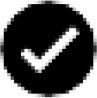 **f**	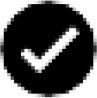				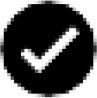	
			N	N									
Lorca (2023) Chile [100]			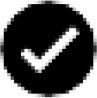			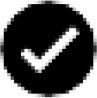	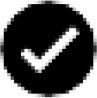 **g**					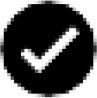	
			Y	Y									
Moorthy (2023) UK [99]	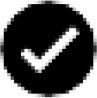		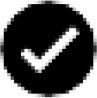			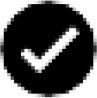	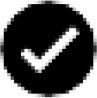 **h**	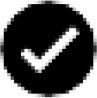		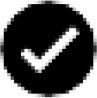	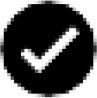 **j**	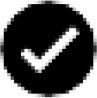	
			Y	Y									
Piche (2023) Canada [101]			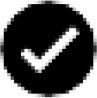			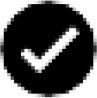	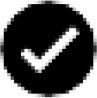 **i**	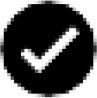		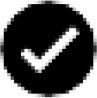		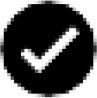	
			Y	Y									
Wu (2021) UK [16]			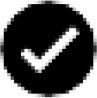			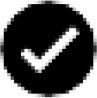		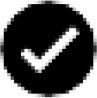		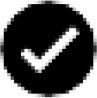	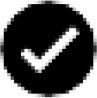 **k**	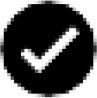	
			Y	N									
Czech (2023) Poland [92]					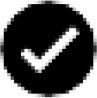					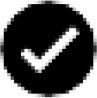	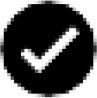 **l**		

Interventions assessed to be effective on a general level, if there is significant improvement post-intervention. 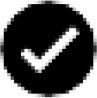 will be assigned for statistically significant improvement in appropriate domain. 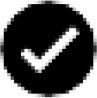 will not be assigned in the effectiveness if details of the particular intervention were not available. Significant improvement in EQ5D with reduced LOS^a^. Significant participation in vigorous physical activity & improvement in 6MWT distance^b^. Significant improvement in shuttle walk test distance^c^. Significant improvement in FACIT-F, FACT-G, HADS^d^. Demonstrated significant increase in stepcount^e.^ Significant improvement in 30secSTST^f^. Significant improvements in BFI, FSTST & STS 1-min^g^. Significant improvement in STS^h^. Significant improvements in 2-min step test & 30secSTST^i^. Significant reduction in distress^j^. Significant improvements in FACIT-F & EQVAS^k^. Significant reduction in distress & anxiety with an increase in coping mechanism^l^.

There was only 1 study on the the use of virtual reality therapy in helping mood and distress (92). The Virtual Reality Garden was applied through VR goggles, providing intense visual, auditory and kinestheic stimuli targeted at producing a calming effect with mood elevation. A variety of cancer patients were involved in all these studies involving technology-enabled prehabilitation.

### Outcomes

#### Primary Outcomes

##### Functional

Physical functional outcomes reported were heterogenous, with 6MWT (91, 94), the incremental shuttle walk (89), sit-to-stand test (STST) (99–101), five-times STST (5STST) (100), 2 min step test (101), heart rate recovery (HRR) (99), step count (93, 99), all reported.

There is a trend towards a lower proportion of improvement in the physical domain when wearables are deployed as the sole technological enabler. Physical intervention may translate into improvements in the psychological and quality of life domains without mental health interventions (95, 100) (**Table 4**).

##### Psychological

Changes in HADS (91, 95), Emotional Distress Scale (99), and Beck Depression Inventory (92) have been reported.

Virtual reality therapy resulted in a significant reduction in the amount of distress post-intervention but did not show a significant difference between the intervention and control groups (92).

A study that involved exercise as the sole mode of intervention resulted in improvements in HADS (95). A study that provided psychological support through a coach did so through a video-conferencing platform and that resulted in a significant drop in distress (99).

##### Quality of life and others

QOL-EORTC (89, 96, 101), FACIT-F (16, 95), FACT-G (95), EQ-5D (16, 90) and BFI (100) were reported.

In the prehabilitation studies that targeted mental health, there were improvements in the FACIT-F and EQVAS (16). Prehabilitation that mainly targeted exercise also resulted in improvements in BFI, FACIT-F and FACT-G (95).

#### Secondary Outcomes

Length of stay (LOS) (90, 99) and postoperative complications (90, 93, 99) were studied. There was a reduction in the length of stay in a controlled study that intervened in exercise, diet, smoking cessation, and breathing exercises for lung cancer. In the 3 studies that reported postoperative complication rates, no differences were found.

Data pertaining to funding for the studies were extracted (**Table 3**). The funding sources were mostly academic or government institutions, or philanthropy. None of these were industrially sponsored.

Quantitative synthesis was not performed as none of the studies were sufficiently powered to inform the efficacy/effectiveness conclusions. Consequently, we were unable to perform a meta-analysis.

### Adverse events

These studies reported no significant adverse events (AEs).

### Feasibility and acceptability

Twelve of the 16 studies discussed the feasibility of conducting these studies (**Table 3**). All of these have been reported to be feasible. Generally, recruitment or attendance was above or close to 70%. Notably, the study population size was generally small, with the majority consisting of 30 patients or fewer (range, 11–100). One study reported more exercise sessions in the application group than in the conventional group despite a shorter wait time to surgery (89). One unintentional convenience sample reported 54% completion and 53% retention, deemed feasible but raising potential selection bias concerns (100). Another also raised concerns due to high drop-out rate of 48% with reasons of inadequate step data obtained, withdrawal and protocol deviation (93). There was acceptance by the participants in the studies, as indicated by patient satisfaction. One study reported the acceptance of technology by the data received (102). However, the patients provided feedback on the fitness device itself, some of which were negative.

### Challenges in implementation

Challenges identified from various studies fell into three categories: technical barriers, lack of direct supervision, and non-compliance.

#### Technical barriers

Some participants in one study cited a self-perceived lack of digital ability and literacy (16). However, this was not a significant barrier to participation in the study. The absence of group sessions was cited as a reason for the lack of opportunities for peer support. Another study reported challenges such as patients requiring assistance for the use of technology, the multidisciplinary team needing to adjust to new technologies, loss to follow-up, and extra costs involved in the use of technology (98). One study reported that some patients were excluded from the study for reasons such as inability to operate a smart phone (97).

#### Lack of direct supervision

A possible lower quality in the execution of exercises owing to their unsupervised nature was a concern (96).

#### Non-compliance

One study faced the challenge of missing data, which could be device malfunction versus a choice not to wear, charge, or synchronize the device or an inability to operate the device (102). Similarly, in another study, patients failed to put on the wearable device daily, thus underestimating the activity level of the participants (94).

## Discussion

This systematic review evaluates the feasibility and effectiveness of technology-based cancer prehabilitation programs, offering recommendations for future research and practice.

### Feasibility

Twelve of sixteen studies evaluated feasibility and all 12 of them reported feasibility, although one raised concerns due to low completion and retention rates (100). Challenges included adapting face-to-face programs to remote delivery during the pandemic without proper piloting (99). Technical issues included wearable-app synchronization failures (102), device malfunctions (102), and user-friendliness concerns (98). Early prototype testing with team members or healthy volunteers is recommended to identify and address technical issues before implementation with cancer patients. Remote prehabilitation’s lack of supervision and compliance might be addressed through teleconferencing for individual or group support (101).

### Determinants of effectiveness

Two key factors contribute to program effectiveness:

1. Intervention Rigor: Effective prehabilitation interventions form the foundation for success. For example, an individualized, structured program adhering to FITT principles with additional physical activity encouragement resulted in significant 6MWT improvement compared to controls (91). Conversely, programs lacking specific exercise prescription and delayed telephonic supervision (93) showed no difference in hospital complication rates. The intervention’s rigor and fidelity significantly impact its success (100).
2. Technology’s Nature and Effectiveness: Wearable technology offers advantages like remote monitoring of step count and heart rate zones, objectively quantifying exercise intensity and frequency without direct supervision. However, disadvantages include potential compliance issues due to lack of supervision and data capture failures. Wearables with real-time feedback to healthcare teams for daily audits and patient feedback during technical disruptions or non-compliance can address these concerns. Videoconferencing can further mitigate compliance issues by providing supervised individual or group exercise sessions (101). Real-time videoconferencing has been accepted as an effective and non-inferior alternative to face-to-face delivery of postsurgical rehabilitation (103) and has been effective in delivering nutritional and psychological counselling (104, 105). The results are seen in one of the studies in our review (99) and its utility should be increased.

### Assessments and outcome measures

Remote prehabilitation’s lack of physical contact limits physical outcome measure collection. One study conducted the first outcome measure in-person and subsequent ones virtually (100). Others opted for virtual assessments only, measuring quality of life outcomes instead (90). One study solely employed virtual physical assessments (99). It is recommended that outcome measures align with the intervention mode and that further validation of virtual physical assessments be conducted for program pragmatism and timely implementation.

### Multimodal prehabilitation

Limited information exists regarding nutritional, mental health, and educational interventions in most studies, except for a few (16, 96). This is concordant with conventional multimodal prehabilitation studies. Additionally, outcome measures related to nutrition, like serum albumin, muscle mass, and handgrip strength, were not commonly reported, except for frailty scores (96). These omissions might be due to the lack of face-to-face consultations. However, ensuring interventions follow established conventional protocols might address this issue, avoiding the need for entirely new intervention designs. Designs can then be tweaked to address the lack of physical contact thereafter.

### An all-in-one system

**Figure 2:**
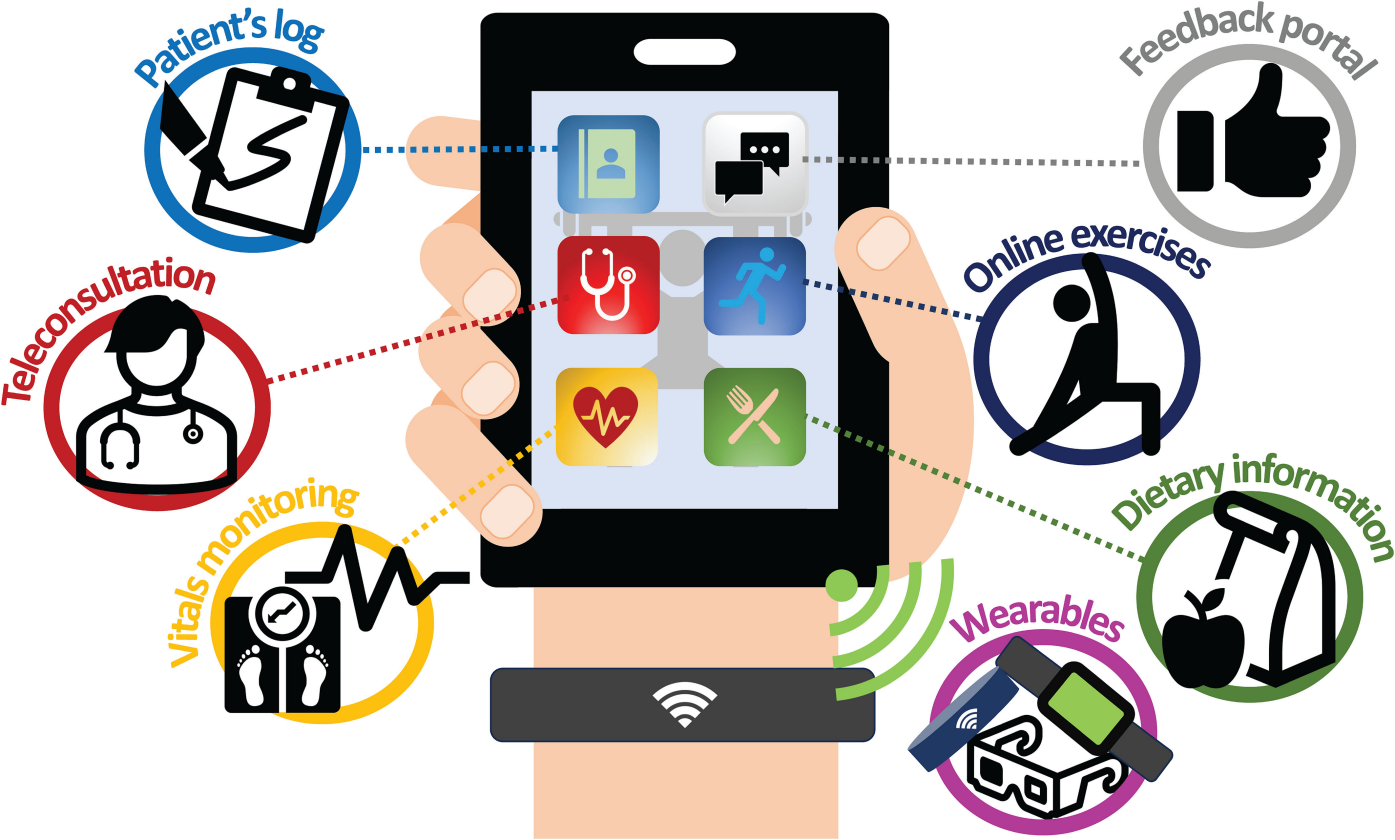
All-in-one system. This figure represents our authors’ impression of an all-in-one system, where patients can easily gain access to a plethora of services at the touch of a screen from a common handheld device. The authors envisioned a virtual platform with the ability to allow teleconsultation/feedback with physicians or allied health workers, either synchronous or asynchronous (provider and user do not have to be online simultaneously to interact), or allowing online exercises to be conducted in a supervised manner in either a 1-on-1 or group setting. In addition, information on diet, psychological well-being, exercise videos and other materials will also be made readily accessible for patients on this platform. This device will also be platform-agnostic as well as Bluetooth and Wi-Fi compatible, allowing seamless syncing and loading of software across many different devices and wearables. Other possibilities for this device include the ability to monitor vital signs, activity/exercise and these information gathered can be successfully shared selectively between user and healthcare providers to allow for server-side analytics.

Based on identified challenges like supervision, compliance, fidelity, and device usability, and insights from large-scale virtual cancer rehabilitation programs (106), remote prehabilitation might benefit from integrating multiple technologies instead of relying on single devices, especially for multimodal programs. Therefore, an “all-in-one system” concept was developed based on the reviewed studies. This system aims to combine features from various studies, potentially offering recorded exercise videos, educational and nutritional guides, teleconferencing capabilities, exercise output measurements, and two-way communication channels. Ideally, this would be a user-friendly, intuitive, and device-agnostic application (**Figure 2**).

The potential of telerehabilitation in specific conditions suggests its applicability to cancer prehabilitation. Notably, studies have successfully implemented telerehabilitation for various conditions, providing valuable insights for its potential use in cancer care. Barriers to accessing traditional prehabilitation programs, such as social isolation, transportation difficulties, limited support networks, inadequate infrastructure, and capacity constraints, can be alleviated with technology-based solutions. Moreover, the looming challenges of healthcare workforce shortages and budgetary limitations, exacerbated by potential future pandemics, highlight the vulnerabilities of traditional models unable to adapt. Challenges can exist for the all-in-one system as well, including technology disruption, necessitating a support team to be accessible. Other challenges include funding and cost of implementation, as well as problems with technology adoption. However, embracing technological innovation and leveraging its capabilities may be a viable path forward for developing accessible and sustainable cancer prehabilitation programs in the future.

### Limitations of the review

This review acknowledges several methodological limitations:

Inclusion of diverse study types: Unlike typical systematic reviews often focused on controlled trials, this review included a broader range of studies due to the preliminary finding of limited high-quality evidence. While this approach ensures comprehensiveness, it may compromise the strength of conclusions drawn.

Choice of quality assessment tools: Employing the NIH tools instead of more widespread options like PEDro or Cochrane RoB 2 facilitated evaluation of diverse study types but may limit comparability with other reviews.

Arbitrary quality score cut-offs: The arbitrary thresholds of 50% and 80% for “good” and “poor” ratings, while informed by previous research, lack formal justification and may require refinement.

We anticipate that future iterations of this review, as the field generates more robust evidence, will be able to address these limitations and provide more definitive conclusions.

## Authors’ conclusions

This systematic review investigated the feasibility and effectiveness of technology-based cancer prehabilitation programs. While the studies included were diverse in quality and design, they collectively offer valuable insights for future research and practice. All studies reported feasibility, although challenges related to technical issues, lack of supervision, and non-compliance were identified. Due to the diversity of the studies, no firm conclusion on effectiveness can be made, although trends are observed. Effectiveness depends on intervention rigor and the nature of the technology employed. Multimodal programs and outcome measures aligned with the intervention mode are crucial. An “all-in-one system” integrating various technologies may be promising for addressing identified challenges. Integrating teleprehabilitation strategies holds potential for wider applicability.

As the field matures, future reviews are expected to provide more definitive conclusions. Embracing technological innovation can pave the way for developing accessible and sustainable cancer prehabilitation programs.

## Data availability statement

The original contributions presented in the study are included in the article/**Supplementary Material**, further inquiries can be directed to the corresponding author.

## Author contributions

ST: Writing – original draft, Writing – review & editing, Conceptualization, Methodology, Resources. FZ: Writing – original draft, Writing – review & editing. EN: Conceptualization, Formal analysis, Writing – original draft, Writing – review & editing.
