# Supplementary material for: The use of technology in cancer prehabilitation: a systematic review
Source: Front Oncol. 2024 Apr 19;14:1321493. doi: 10.3389/fonc.2024.1321493 (PMC11066209; doi:10.3389/fonc.2024.1321493)
Supplement: Supplementary file 1 [file Table_1.docx]

**Supplementary Table 1. List of excluded full text articles**

| **Failed Criteria #1**  **Nil involvement of primary research (randomised and non-randomised experimental trials, cohort or case-control studies, as well as case series and case reports))** | |
| --- | --- |
| 1 | Rammant E, Deforche B, Va Hecke A et al. Development of a pre-and postoperative physical activity promotion integrated in the electronic health system of patients with bladder cancer (The POPEYE study): An intervention mapping approach. Eur J Cancer Care (Engl). 2021 Mar;30(2): e13363. Doi: 10.1111/ecc.13363. Epub 2020 Nov 17. |
| 2 | Sell NM, Silver JK, Rando S, Draviam AC, Mina DS, Qadan M. Prehabilitation Telemedicine in Neoadjuvant Surgical Oncology Patients During the Novel COVID-19 Coronavirus Pandemic. Ann Surg (2020) Aug;272(2): e81-e83. |
| 3 | Wu F, Laza-Cagigas R, Rampal T. Understanding Patient’s Experiences and Perspectives of Tele-Prehabilitation: A Qualitative Study to Inform Study Design and Delivery. Clin Pract. 2022 Aug 16;12(4):640-652. Di:10.3390/clinpract12040067 |
| 4 | Lambert G, Drummond K, Ferreira V, Carli F. Teleprehabilitation during COVID-19 pandemic: the essentails of “what” and “how”. Support Care Cancer. 2021 Feb;29(2):551-554. Doi:10.1007/s00520-020-05768-4. Epub 2020 Sep12. |
| 5 | Durrand JW, Moore J, Danjoux G. Prehabilitation and preparation for surgery: has the digital revolution arrived? Anaesthesia. 2022 Jun;77(6): 635-639. Doi:10.1111/anae.15622. Epub 2021 Nov 18. |
| 6 | Gonella F, Massucco P, Perotti S, Monasterolo S, Vassallo D, Laezza A, et al. Telemedicine prehabilitation as a result of COVID-19: disruptive technological solutions. Br J Surg (2021) Jun 22;108(6): e215-e216. doi: 10.1093/bjs/znab066 |
| 7 | Steffans D, Delbaere K, Young J, Solomon M, Denehy L. Evidence on technology-driven preoperative exercise interventions: are we there yet? Br J Anaesth. 2020Nobv;125(5):646-649. Doi: 10.1016/j.bja.2020.06.050. Epiub 2020 Jul 15. |
| 8 | Asberg K, Bendtsen Perioperative digital behaviour change interventions for reducing alcohol consumption, improving dietary intake, increasing physical activity and smoking cessation: a scoping review. Perioper Med (Lond). 2021 Jul 6;10(1):18. Doi: 10.1186/s13741-021-00189-1. |
| 9 | Barberan-Garcia A, Cano I, Bongers BC, Seyfried S, Ganslandt T, Herrle F, et al. Digital Support to Multimodal Community- Based Prehabilitation: Looking for Optimization of Health Value Generation. Front Oncol (2021) Jun 17; 11:662013. doi: 10.3389/fonc.2021.662013 |
| 10 | Kelly AE. Telehealth: only a click away. Journal of Psychosocial Oncology (2021) 39:3(337-339) |
| 11 | Townsend, William Blair, Worrilow et al. The benefit of prehabilitation and enhanced recovery in robot-assisted radical prostatectomy and the promising future of these protocols in the field of urologic oncology. Cancer. 2020 Sep 15;126(18):4107-4109. doi: 10.1002/cncr.33059. Epub 2020 Jul 8 |
| 12 | Huston A. Development of Virtual Integrative Oncology Centre. Oncology Issues (2022) 37:2(38-45) |
| 13 | Robinson A, Oksuz U, Slight R et al. Digital and Mobile Technologies to promote Physical Health Behaviour Change and Provide Psychological Support for Patients Undergoing Elective Surgery: Meta-Ethnography and Systematic Review. JMIR Mhealth Uhealth.2020 Dec1;8(12): e19237. Doi:10.2196/19237. |
| 14 | Karlsson E, Dahl O, Rydwik E, Nygren-Bonnier M, Bergenmar M. Older patient’s attitudes towards, and perceptions of, preoperative physical activity and exercise prior to colorectal cancer surgery- a gap between awareness and action. Supportive Care Cancer. 2020 Aug;28(8): 3945-3953. Doi:10.1007/s00520-019-05237-7. Epub 2019 Dec 20. |
| 15 | Seery T, Wu F, Hendricks E et al. Patients, experiences of virtual prehabilitation during the COVID-19 pandemic. Patient Education and Counselling (2022) 105:2 (488-489) |
| 16 | Li T.-C., Yang M.-C, Tseng A.H et al. Prehabilitation and rehabilitation for surgically treated lung cancer patients. Journal of Cancer Research and Practice (2017) 4;3 (89-94). |
| 17 | Zhou YB. Prehabilitation for gastrointestinal cancer patients. Zhonghua Wei Chang Wai Ke Za Zhi. 2021 Feb25;24(2):122-127. Doi:10.3760/cma.j.cn.441530-20200318-00152. |
| 18 | Waterland, JL, Chahal R, Ismail H, Sinton C, Riedel B, Francis JJ, et al. Implementing a telehealth prehabilitation education session for patients preparing for major cancer surgery. BMC Health Serv Res (2021) 21:443. doi: 10.1186/s12913-021-06437-w |
| 19 | Machado PFA, Oliveiros B, Martins RA, Cruz J. ASO Author Reflections: Impact of a Preoperative Home-Based Exercise Program on Quality of Life After Lung Cancer Resection. Ann Surg Oncol. 2024 Feb;31(2):897-898. doi: 10.1245/s10434-023-14620-y. Epub 2023 Nov 19. PMID: 37980706. |
| 20 | Neuendorf T, Haase R, Schroeder S, Schumann M, Nitzsche N. Effects of high-intensity interval training on functional performance and maximal oxygen uptake in comparison with moderate intensity continuous training in cancer patients: a systematic review and meta-analysis. Support Care Cancer. 2023 Oct 18;31(12):643. doi: 10.1007/s00520-023-08103-9. PMID: 37851104; PMCID: PMC10584719. |
| 21 | Guo Y, Ding L, Miao X, Jiang X, Xu T, Xu X, Zhu S, Xu Q, Hu J. Effects of prehabilitation on postoperative outcomes in frail cancer patients undergoing elective surgery: a systematic review and meta-analysis. Support Care Cancer. 2022 Dec 19;31(1):57. doi: 10.1007/s00520-022-07541-1. PMID: 36534300. |
| 22 | Edbrooke L, Bowman A, Granger CL, Burgess N, Abo S, Connolly B, Denehy L. Exercise across the Lung Cancer Care Continuum: An Overview of Systematic Reviews. J Clin Med. 2023 Feb 27;12(5):1871. doi: 10.3390/jcm12051871. PMID: 36902659; PMCID: PMC10003899. |
| 23 | Christopher CN, Kang DW, Wilson RL, Gonzalo-Encabo P, Ficarra S, Heislein D, Dieli-Conwright CM. Exercise and Nutrition Interventions for Prehabilitation in Hepato-Pancreato-Biliary Cancers: A Narrative Review. Nutrients. 2023 Dec 8;15(24):5044. doi: 10.3390/nu15245044. PMID: 38140303; PMCID: PMC10745391. |
| 24 | Gillman A, Hayes M, Sheaf G, Walshe M, Reynolds JV, Regan J. Exercise-based dysphagia rehabilitation for adults with oesophageal cancer: a systematic review. BMC Cancer. 2022 Jan 10;22(1):53. doi: 10.1186/s12885-021-09155-y. PMID: 35012495; PMCID: PMC8751332. |
| 25 | Parraguez LAL, Ribeiro IL, Hinojosa MP, Troncoso JP. Implementation of a teleprehabilitation program for oncosurgical patients during the COVID-19 pandemic: perspectives and user satisfaction. Support Care Cancer. 2023 May 22;31(6):346. doi: 10.1007/s00520-023-07799-z. PMID: 37212973; PMCID: PMC10201043. |
| 26 | Whish-Wilson GA, Edbrooke L, Cavalheri V, Denehy L, Seller D, Granger CL, Parry SM. Physiotherapy and Exercise Management of People Undergoing Surgery for Lung Cancer: A Survey of Current Practice across Australia and New Zealand. J Clin Med. 2023 Mar 9;12(6):2146. doi: 10.3390/jcm12062146. PMID: 36983146; PMCID: PMC10051547. |
| 27 | Raff C, Dörr-Harim C, Otto S, Thiele J, Mihaljevic A, Kramer K. Prehabilitation in an Integrative Medicine Day Clinic for Patients Undergoing Neoadjuvant Treatment: Single-Center Feasibility Pilot Study. JMIR Res Protoc. 2023 Oct 18;12:e46765. doi: 10.2196/46765. PMID: 37851493; PMCID: PMC10620634. |
| 28 | Flores LE, Westmark D, Katz NB, Hunter TL, Silver EM, Bryan KM, Jagsi R, McClelland S 3rd, Silver JK. Prehabilitation in radiation therapy: a scoping review. Support Care Cancer. 2024 Jan 5;32(1):83. doi: 10.1007/s00520-023-08262-9. PMID: 38177946. |
| 29 | Meneses-Echavez JF, Loaiza-Betancur AF, Díaz-López V, Echavarría-Rodríguez AM, Triana-Reina HR. Prehabilitation programs for individuals with cancer: a systematic review of randomized-controlled trials. Syst Rev. 2023 Nov 17;12(1):219. doi: 10.1186/s13643-023-02373-4. PMID: 37978411; PMCID: PMC10655304. |
| 30 | Rozenberg D. Rehabilitation pre- and post thoracic surgery: Progress and future opportunities. Chron Respir Dis. 2023 Jan-Dec;20:14799731231165305. doi: 10.1177/14799731231165305. PMID: 36941268; PMCID: PMC10031604. |
| 31 | Lippi L, Turco A, Moalli S, Gallo M, Curci C, Maconi A, de Sire A, Invernizzi M. Role of Prehabilitation and Rehabilitation on Functional Recovery and Quality of Life in Thyroid Cancer Patients: A Comprehensive Review. Cancers (Basel). 2023 Sep 10;15(18):4502. doi: 10.3390/cancers15184502. PMID: 37760472; PMCID: PMC10526253. |
| 32 | Jurys T, Kupilas A, Rajwa P, Bryniarski P, Burzyński B. Role of preoperative patient education among prostate cancer patients treated by radical prostatectomy. Cent European J Urol. 2022;75(3):272-276. doi: 10.5173/ceju.2022.0037. Epub 2022 Aug 18. PMID: 36381162; PMCID: PMC9628718. |
| 33 | Mareschal J, Hemmer A, Douissard J, Dupertuis YM, Collet TH, Koessler T, Toso C, Ris F, Genton L. Surgical Prehabilitation in Patients with Gastrointestinal Cancers: Impact of Unimodal and Multimodal Programs on Postoperative Outcomes and Prospects for New Therapeutic Strategies-A Systematic Review. Cancers (Basel). 2023 Mar 21;15(6):1881. doi: 10.3390/cancers15061881. PMID: 36980767; PMCID: PMC10047365. |
| 34 | Raz DJ, Kim JY, Erhunwmunesee L, Hite S, Varatkar G, Sun V. The value of perioperative physical activity in older patients undergoing surgery for lung cancer. Expert Rev Respir Med. 2023 Jul-Dec;17(8):691-700. doi: 10.1080/17476348.2023.2255133. Epub 2023 Sep 11. PMID: 37668168. |
| 35 | McCann L, Hewitt C, McMillan KA. Developing an e-Prehabilitation System of Care for Young Adults Diagnosed With Cancer: User-Centered Design Study. JMIR Cancer. 2023 Mar 30;9:e41441. doi: 10.2196/41441. PMID: 36995740; PMCID: PMC10131730. |
| 36 | Cooper M, Chmelo J, Sinclair RCF, Charman S, Hallsworth K, Welford J, Phillips AW, Greystoke A, Avery L. Exploring factors influencing uptake and adherence to a home-based prehabilitation physical activity and exercise intervention for patients undergoing chemotherapy before major surgery (ChemoFit): a qualitative study. BMJ Open. 2022 Sep 22;12(9):e062526. doi: 10.1136/bmjopen-2022-062526. PMID: 36137639; PMCID: PMC9511537. |
| 37 | Hunter H, Bennington-McKay N, Sher J, Psutka SP, Lin C. Emerging Role of Mobile Applications and Wearable Devices for Prehabilitation in Urologic Oncology. Eur Urol Focus. 2023 Nov 1:S2405-4569(23)00227-4. doi: 10.1016/j.euf.2023.10.010. Epub ahead of print. PMID: 37923631. |
| 38 | Steffens D, Denehy L, Solomon M, Koh C, Ansari N, McBride K, Carey S, Bartyn J, Lawrence AS, Sheehan K, Delbaere K. Consumer Perspectives on the Adoption of a Prehabilitation Multimodal Online Program for Patients Undergoing Cancer Surgery. Cancers (Basel). 2023 Oct 18;15(20):5039. doi: 10.3390/cancers15205039. PMID: 37894406; PMCID: PMC10605909. |
| 39 | van Deursen L, van der Vaart R, Alblas EE, Struijs JN, Chavannes NH, Aardoom JJ. Improving the colorectal cancer care pathway via e-health: a qualitative study among Dutch healthcare providers and managers. Support Care Cancer. 2023 Mar 6;31(4):203. doi: 10.1007/s00520-023-07653-2. PMID: 36872396; PMCID: PMC9986036. |
| 40 | Ip N, Zhang K, Karimuddin AA, Brown CJ, Campbell KL, Puyat JH, Sutherland JM, Conklin AI. Preparing for colorectal surgery: a feasibility study of a novel web-based multimodal prehabilitation programme in Western Canada. Colorectal Dis. 2024 Jan 16. doi: 10.1111/codi.16851. Epub ahead of print. PMID: 38229235. |
| **Failed Criteria #2**  **Nil involvement of cancer prehabilitation as an intervention** | |
| 1 | Granger CL, Irving L, Antippa P et al. CAPACITY: A physical activity self-management program for patients undergoing surgery for lung cancer, a phase 1 feasibility study. Lung Cancer. 2018 10.1016/j.lungcan.2018.07.034.Epub 2018Jul 23. |
| 2 | Cuadros L, Ismail H, Ho K. Evaluation of Reliability of MYZONE MZ-3 Heart Rate Monitor: A Study for the future of Telephysiotherapy for preoperative Prehabilitation in Cancer Patients. Tele JE Health. 2017 Apr;23(4):334-338. Doi: 10.1089/tmj.2016.0138.Epub 2016 Aug 18. |
| 3 | Heidy Cos, Jorge G Zárate Rodríguez, Rohit Srivastava et al. 4,300 steps per day prior to surgery are associated with improved outcomes after pancreatectomy. HPB (Oxford). Oct; 124: 102-109. Doi: 2023 Jan;25(1):91-99. doi: 10.1016/j.hpb.2022.09.011. Epub 2022 Oct 1. |
| 4 | Amari T, Matta D, Makita Y, Fukuda K, Miyasaka H, Kimura M, Sakamoto Y, Shimo S, Yamaguchi K. Early Ambulation Shortened the Length of Hospital Stay in ICU Patients after Abdominal Surgery. Clin Pract. 2023 Dec 18;13(6):1612-1623. doi: 10.3390/clinpract13060141. PMID: 38131690; PMCID: PMC10742920. |
| 5 | Chen, C. C. G., Malpani, A., Waldram, M. M., Romanczyk, C., Tanner, E. J., Fader, A. N., Scheib, S. A., Hager, G. D., & Vedula, S. S. (2023). Effect of pre-operative warm-up on trainee intraoperative performance during robot-assisted hysterectomy: a randomized controlled trial. *International Urogynecology Journal*, *34*(11), 2751-2758. https://doi.org/10.1007/s00192-023-05595-1 |
| 6 | Wu Y, Wang X, Gao F, Liao J, Zeng J, Fan L. Mobile nutrition and health management platform for perioperative recovery: an interdisciplinary research achievement using WeChat Applet. Front Med (Lausanne). 2023 May 24;10:1201866. doi: 10.3389/fmed.2023.1201866. PMID: 37293309; PMCID: PMC10244757. |
| 7 | Alverdy JC. Rationale for Colonic Pre-Habilitation Prior to Restoration of Gastrointestinal Continuity. Surg Infect (Larchmt). 2023 Apr;24(3):265-270. doi: 10.1089/sur.2023.001. PMID: 37010975; PMCID: PMC10061335. |
| **Failed Criteria #4**  **Nil mention of utilised technology such as trackers, apps, telehealth, virtual or online platforms and robotic devices for cancer prehabilitation** | |
| 1 | van Gestel T, Groen LCB, Puik JR, van Rooijen SJ, van der Zaag-Loonen HJ, Schoonmade LJ, Danjoux G, Daams F, Schreurs WH, Bruns ERJ. Fit4Surgery for cancer patients during covid-19 lockdown - A systematic review and meta-analysis. Eur J Surg Oncol. 2022 Feb 9:  S0748-7983(22)00085-3. doi: 10.1016/j.ejso.2022.02.010 |
| 2 | Steffans D, Solomon M, Denehy L. Is preoperative exercise training the new holy grail for patients undergoing major surgery? Annals of the American Thoracic Society (2021) 18:4(587-589) |
| 3 | Carli F, Scheede-Bergdahl C. Prehabilitation to Enhance Perioperative Care. Anesthesiology Clinics (2015) 33:1(17-33) |
| 4 | Bingham SL, Small S, Semple CJ (2023) A qualitative evaluation of a multi-modal cancer prehabilitation programme for colorectal, head and neck and lung cancers patients. PLoS ONE 18(10): e0277589. https://doi.org/10.1371/journal.pone.0277589 |
| 5 | Machado P, Pimenta S, Garcia AL, Nogueira T, Silva S, Dos Santos CL, Martins MV, Canha A, Oliveiros B, Martins RA, Cruz J. Effect of Preoperative Home-Based Exercise Training on Quality of Life After Lung Cancer Surgery: A Multicenter Randomized Controlled Trial. Ann Surg Oncol. 2024 Feb;31(2):847-859. doi: 10.1245/s10434-023-14503-2. Epub 2023 Nov 7. PMID: 37934383; PMCID: PMC10761542. |
| 6 | Molenaar CJL, Minnella EM, Coca-Martinez M, et al. Effect of Multimodal Prehabilitation on Reducing Postoperative Complications and Enhancing Functional Capacity Following Colorectal Cancer Surgery: The PREHAB Randomized Clinical Trial. *JAMA Surg.* 2023;158(6):572–581. doi:10.1001/jamasurg.2023.0198 |
| 7 | Akdemir E, Sweegers MG, Vrieling A, Rundqvist H, Meijer RP, Leliveld-Kors AM, van der Heijden AG, Rutten VC, Koldewijn EL, Bos SD, Wijburg CJ, Marcelissen TAT, Bongers BC, Retèl VP, van Harten WH, May AM, Groen WG, Stuiver MM. EffectiveNess of a multimodal preHAbilitation program in patieNts with bladder canCEr undergoing radical cystectomy: protocol of the ENHANCE multicentre randomised controlled trial. BMJ Open. 2023 Mar 7;13(3):e071304. doi: 10.1136/bmjopen-2022-071304. PMID: 36882246; PMCID: PMC10008243. |
| 8 | McCourt O, Fisher A, Ramdharry G, Land J, Roberts AL, Rabin N, Yong K. Exercise prehabilitation for people with myeloma undergoing autologous stem cell transplantation: results from PERCEPT pilot randomised controlled trial. Acta Oncol. 2023 Jul;62(7):696-705. doi: 10.1080/0284186X.2023.2178326. Epub 2023 Feb 15. PMID: 36794394. |
| 9 | Bradley P, Merchant Z, Rowlinson-Groves K, Taylor M, Moore J, Evison M. Feasibility and outcomes of a real-world regional lung cancer prehabilitation programme in the UK. Br J Anaesth. 2023 Jan;130(1):e47-e55. doi: 10.1016/j.bja.2022.05.034. Epub 2022 Jul 13. PMID: 35840361; PMCID: PMC9875904. |
| 10 | Finch A., Assadourian A., Grant D., Redman J., Jalali S., Ricketts W. Impact of prehabilitation on pre-operative function, post-operative complications and length of stay in resectable lung cancers. Lung Cancer (2023) 178 Supplement 1 (S82-S83). http://dx.doi.org/10.1016/S0169-5002(23)00614-1 |
| 11 | Smits A, Agius CM, Blake D, Ang C, Kucukmetin A, Ham MV, Pijnenborg JMA, Knight J, Rundle S. Is Cardiopulmonary Exercise Testing Predictive of Surgical Complications in Patients Undergoing Surgery for Ovarian Cancer? Cancers (Basel). 2023 Oct 28;15(21):5185. doi: 10.3390/cancers15215185. PMID: 37958358; PMCID: PMC10648080. |
| 12 | Strijker D, Meijerink WJHJ, van Heusden-Schotalbers LAG, van den Berg MGA, van Asseldonk MJMD, Drager LD, de Wilt JHW, van Laarhoven KJHM, van den Heuvel B. Multimodal Prehabilitation in Patients Undergoing Complex Colorectal Surgery, Liver Resection, and Hyperthermic Intraperitoneal Chemotherapy (HIPEC): A Pilot Study on Feasibility and Potential Efficacy. Cancers (Basel). 2023 Mar 20;15(6):1870. doi: 10.3390/cancers15061870. PMID: 36980756; PMCID: PMC10047129. |
| 13 | Wu J, Chi H, Kok S, Chua JMW, Huang XX, Zhang S, Mah S, Foo LX, Peh HY, Lee HB, Tay P, Tong C, Ladlad J, Tan CHM, Khoo N, Aw D, Chong CXZ, Ho LML, Sivarajah SS, Ng J, Tan WJH, Foo FJ, Teh BT, Koh FH. Multimodal prerehabilitation for elderly patients with sarcopenia in colorectal surgery. Ann Coloproctol. 2023 Mar 31. doi: 10.3393/ac.2022.01207.0172. Epub ahead of print. PMID: 37004990. |
| 14 | Christodoulidis G, Halliday LJ, Samara A, Bhuva N, Park WE, Moorthy K. Personalized Prehabilitation Improves Tolerance to Chemotherapy in Patients with Oesophageal Cancer. Curr Oncol. 2023 Jan 24;30(2):1538-1545. doi: 10.3390/curroncol30020118. PMID: 36826079; PMCID: PMC9955115. |
| 15 | Sole-Sedeno JM, Miralpeix E, Muns MD, Rodriguez-Cosmen C, Fabrego B, Kanjou N, Medina FX, Mancebo G. Protein Supplementation in a Prehabilitation Program in Patients Undergoing Surgery for Endometrial Cancer. Int J Environ Res Public Health. 2023 Apr 13;20(8):5502. doi: 10.3390/ijerph20085502. PMID: 37107783; PMCID: PMC10139161. |
| 16 | Kilinc F, Setzer M, Prinz V, Jussen D, Marquardt G, Gessler F, Czabanka M, Freiman T, Dubinski D, Won SY, Haberland M, Behmanesh B. The Beneficial Effect of Preoperative Exercise on Postoperative Clinical Outcome, Quality of Life and Return to Work after Microsurgical Resection of Spinal Meningiomas. J Clin Med. 2023 Apr 10;12(8):2804. doi: 10.3390/jcm12082804. PMID: 37109141; PMCID: PMC10146916. |
| 17 | San San Tay. Perspectives on the Direction of Cancer Prehabilitation in the Pandemic and Beyond. Arch Rehabil Res Clin Transl. 2022 Dec;4(4):100236. doi: 10.1016/j.arrct.2022.100236. Epub 2022 Oct 17. |
| 18 | Thoft Jensen B, Bjerggaard Jensen J. One-Year Follow-Up after Multimodal Prehabilitation Interventions in Radical Cystectomy. Cancers (Basel). 2023 Dec 10;15(24):5785. doi: 10.3390/cancers15245785. PMID: 38136331; PMCID: PMC10741904. |
| 19 | Mawson S, Keen C, Skilbeck J et al. Feasibility and benefits of a structured prehabilitation programme prior to autologous stem cell transplantation (ACST) in patients with myeloma; a prospective feasibility study. Physiotherapy. 2021 Dec; 113:88-99. Doi: 10.1016/j.physio.2021.08.001. |
